# A Qualitative Study Exploring Patient, Family Carer and Healthcare Professionals’ Direct Experiences and Barriers to Providing and Integrating Palliative Care for Advanced Head and Neck Cancer

**DOI:** 10.1177/0825859720957817

**Published:** 2020-09-15

**Authors:** Catriona Rachel Mayland, Hannah C. Doughty, Simon N. Rogers, Anna Gola, Stephen Mason, Cathy Hubbert, Dominic Macareavy, Barbara A. Jack

**Affiliations:** 1 Department of Oncology and Metabolism, 7315 University of Sheffield, United Kingdom; 2 Palliative Care Institute, 4591 University of Liverpool, United Kingdom; 3 Department of Primary Care and Mental Health, 4591 University of Liverpool, United Kingdom; 4 Faculty of Health and Social Care, 6249 Edge Hill University, Ormskirk, United Kingdom; 5 89542 Aintree University Hospitals NHS Foundation Trust, Liverpool, United Kingdom; 6 Marie Curie Palliative Care Research Department, 4919 University College London, United Kingdom; 7 429822 Aintree Park General Practice, Liverpool, United Kingdom

**Keywords:** head and neck cancer, palliative care, qualitative research, integrated care, end-of-life care

## Abstract

**Objectives:**

To report on direct experiences from advanced head and neck cancer patients, family carers and healthcare professionals, and the barriers to integrating specialist palliative care.

**Methods:**

Using a naturalistic, interpretative approach, within Northwest England, a purposive sample of adult head and neck cancer patients was selected. Their family carers were invited to participate. Healthcare professionals (representing head and neck surgery and specialist nursing; oncology; specialist palliative care; general practice and community nursing) were recruited. All participants underwent face-to-face or telephone interviews. A thematic approach, using a modified version of Colazzi’s framework, was used to analyze the data.

**Results:**

Seventeen interviews were conducted (9 patients, 4 joint with family carers and 8 healthcare professionals). Two main barriers were identified by healthcare professionals: “lack of consensus about timing of Specialist Palliative Care engagement” and “high stake decisions with uncertainty about treatment outcome.” The main barrier identified by patients and family carers was “lack of preparedness when transitioning from curable to incurable disease.” There were 2 overlapping themes from both groups: “uncertainty about meeting psychological needs” and “misconceptions of palliative care.”

**Conclusions:**

Head and neck cancer has a less predictable disease trajectory, where complex decisions are made and treatment outcomes are less certain. Specific focus is needed to define the optimal way to initiate Specialist Palliative Care referrals which may differ from those used for the wider cancer population. Clearer ways to effectively communicate goals of care are required potentially involving collaboration between Specialist Palliative Care and the wider head and neck cancer team.

## Introduction

Head and neck cancer (HNC) has unique complexities due to its effects on eating, speaking and breathing.^1-3^ This results in prevalent, diverse symptoms^2,4^ with complex pain often experienced.^4^ Issues with speech can cause difficulties with expressing needs, impacting on involvement with decision-making. Altered facial appearance,^5,6^ distressing symptoms and social isolation can contribute to depression and a higher risk of suicide.^7^ Family members report distress and unmet needs.^8,9^ Globally, poverty and deprivation impact on care access.^10,11^

Specialist Palliative Care (SPC) input with HNC patients may be underutilised, despite the improved symptom control and patient experience when SPC is introduced early into oncological care.^12-14^ Within the UK, only 25% of HNC multi-disciplinary team (MDT) meetings have direct SPC presence^15^ although this may relate to meetings being focused on initial diagnosis and treatment plans. In one retrospective, national study, conducted within the U.S.A., only 5% of hospitalized metastatic HNC patients received a SPC consultation.^16^ In other studies, referral for SPC input within the hospital or for hospice care could be late in the course of the disease.^17,18^ Variability in accessing services for age and gender has been seen for HNC patients.^16,18^ More widely, low socio-economic groups experience barriers in access to SPC services,^19^ which is especially pertinent for HNC patients, where socio-economic status is a recognized factor in both incidence and survival.^20,21^

There are many barriers to integrating palliative care into cancer care^22^: lack of oncologists awareness or knowledge about palliative care^23^; lack of effective communication between healthcare professionals and patients (e.g. goals of care)^24^; limited palliative care resources^24^; societal misconceptions about palliative care meaning end-of-life^24^; and lack of sufficient research funding. No studies have explored the specific challenges affecting HNC patients. The aim of this study was to identify the main barriers to integrating SPC within routine oncological care, as perceived by HNC patients, their family carers and healthcare professionals (HCP). The following definition is used for “SPC”: multi-disciplinary teams comprised of individuals who have undertaken specific expert training focused on palliative care needs which cannot be met by patients’ usual healthcare team; SPC teams may operate within the UK hospital, community or hospice setting.

## Methods

We adopted a naturalistic, interpretative approach^25^ to enable a rich understanding of experiences and perceptions.^26^

### Study Setting

Within the UK, HNC care is based on a centralized multidisciplinary model with service integration advocated via a “key worker” role and usually facilitated by a specialist HNC nurse.^27^ National recommendations advise all professionals caring for HNC patients assess palliative and supportive care needs throughout the illness, including at initial treatment planning, and recognize when SPC expertise is required.^28^ A weekly, regional MDT meeting occurs within Northwest England which discusses all new and recurrent HNC patients (average 70 patients/month; 8 treated with palliative intent). Regionally, SPC services, funded through public and charitable sources, provide advisory input to community settings (home, care home or out-patient clinic) working with other generic palliative caregivers e.g. General Practitioners and District Nurses (community doctors and nurses). Advisory SPC input is provided within acute hospitals, working with core professionals from the HNC MDT, or SPC teams are directly responsible for care within a SPC in-patient unit or hospice.

### Participant Selection

#### Patients and family carers

Adult patients (over 18 years) with a histological or radiological diagnosis of “advanced” HNC and aware of their diagnosis (as reported by the clinical team) were purposively sampled.^29^ “Advanced” HNC incorporated those with incurable disease, and those treated curatively but whom the clinical team judged were “high risk” for developing recurrent disease. Those unable to provide informed consent, perceived to be unduly distressed by participation (either by the clinical or research team), or who lived out with the region (and so a face-to-face interview would be burdensome) were excluded. Identification was conducted by clinical teams during HNC MDT meetings, via out-patient clinics and SPC services in hospitals and hospices, who provided initial study information and permission for the research team to make contact. Opportunities for further information and questions were provided. For each patient, where possible, the family carer was asked if they wished to participate.

#### Health care professionals

Potential participants were identified using a “word-of-mouth” snowball sampling strategy which is recognized to benefit “inductive, theory-building analysis.”^29^ Existing linkages with the HNC MDT identified potential community participants. Initially, we aimed to gain views from at least 1 representative working within HNC Surgery; Oncology; SPC; General Practice and Community Nursing. Review of this, deemed that HNC Clinical Nurse Specialist experiences would further enrich the data, in keeping with the concept of “information power,”^30^ which is an alternative approach to the “data saturation” concept. Those wishing to know more about the study, either made direct contact with the research team or passed on their details via existing participants.

### Ethics Approval

Ethical approval was obtained from the Health Research Authority and the North West -Greater Manchester West Research Ethics Committee (REC 17/NW/0083; IRAS project ID 221772). All participants received a Participant Information Sheet and provided written informed consent.

### Data Collection

Patient data were collected from case records and included demographic details; Eastern Cooperative Oncology Group (ECOG) performance status^31^; primary diagnosis; presence of metastatic disease; and treatment intent. Family carer data included gender and relationship to patient. Details for HCPs included gender; age; and time working in current area of practice.

### Interviews

Semi-structured digitally recorded interviews were conducted in a place convenient for the patient (home, hospital or hospice). Either face-to-face or digitally recorded telephone interviews (a logistical, flexible solution to those who work across a wider geographical area)^32^ were offered for HCPs. All interviews were conducted by 1 researcher between June and November 2017. Patient and family carer interviews ranged from 8 to 114 minutes (mean 45 minutes). For 2 patients, verbal communication was especially challenging, so written communication supplemented the interview (and was directly checked with the participant for accuracy). The HCP interviews ranged between 23 and 55 minutes (mean 39 minutes). Field notes were captured immediately after the interviews.

Exploratory topics within the interview schedule (Supplemental File 1) focused on experiences of HNC and specific challenges to integrating SPC. The interview schedule was reviewed and tested by the research team which included medical, nursing, psychology and patient representation.

### Analysis

Demographic data were analyzed descriptively and recorded interviews were transcribed *verbatim* and anonymized during this process. For the 2 patients where verbal communication was challenging, a written record was documented by the researcher. A thematic approach to analysis was conducted using the modified principles of Colazzi’s framework,^33^ namely: organization; familiarization; reduction; and analysis.

To enhance rigor, 2 researchers independently analyzed each transcript to familiarize themselves with the data, recorded initial analytical notes and checked field notes. An inductive approach to coding was used. Both researchers met to compare initial analysis and group the codes together into categories. Data relating to the main research question, the barriers to integrating palliative care, was used as a framework for charting further analysis. Additional discussion, data reduction and analysis across cases was conducted with a third researcher. A final coding scheme was agreed leading to identification of themes and subthemes. All participants were allocated a unique identifier code with participants’ quotes used to support findings. For the 2 patients (P1 and P5), where verbal communication was challenging, written communication was incorporated to supplement the interview, and this is indicated by an asterix (*).

## Results

### Participants

**Figure 1.**
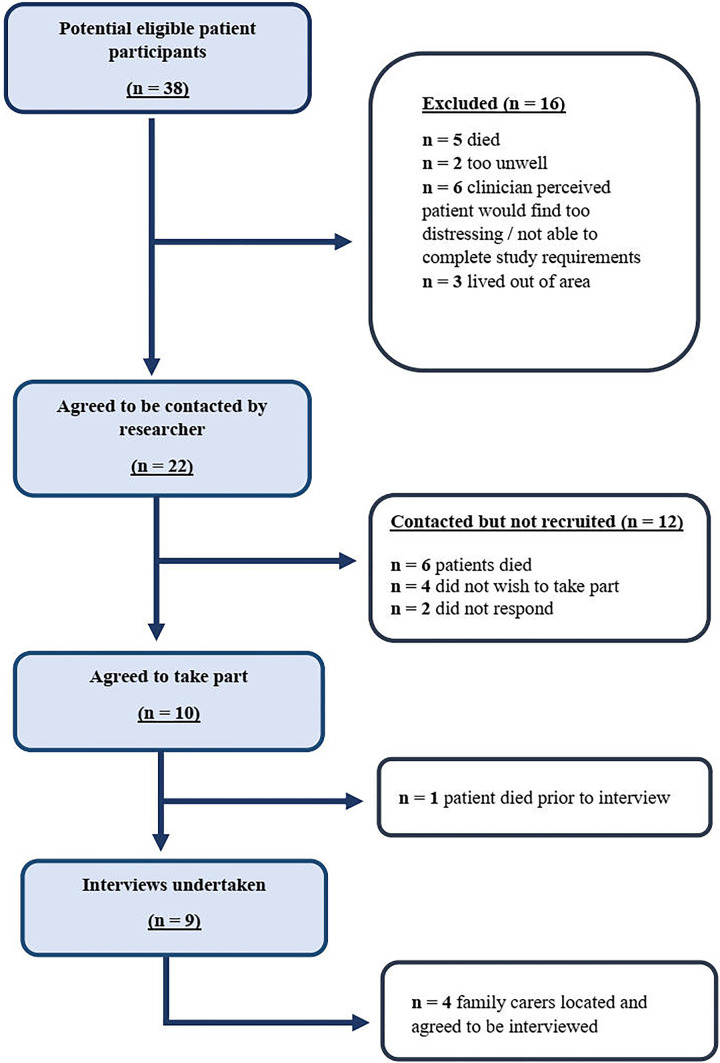
Flow Diagram Illustrating Patient Recruitment.

From 38 eligible patient participants, 10 agreed to be interviewed (Figure 1). One patient died prior to the interview, resulting in a sample of 9 patients. Seven participants were male, and all were aged between 57 and 88 years. There was a wide range of different cancer sites and 4 participants had distant metastatic disease (Table 1).

**Table 1.** Patient and Family Carer Participants’ Demographic and Clinical Details.

Participant	Gender	Age range	ECOG status	Presence of distal metastatic disease*	Treatment intent	Place of care at time of interview	Family carer (FC) interviewed	FC gender	FC relationship to patient
P01	Male	70-79	Not recorded	Yes	Palliative	Hospice	No	N/A	N/A
P02	Male	60-69	2	No	Palliative	Home	Yes (FC2)	Female	Wife
P03	Female	50-59	3	Yes	Palliative	Hospice	No	N/A	N/A
P04	Male	80-89	2	No	Palliative	Hospital	No	N/A	N/A
P05	Male	60-69	1	Yes	Palliative	Hospital	No	N/A	N/A
P06	Male	80-89	1	No	Palliative	Hospital	No	N/A	N/A
P07	Female	60-69	2	Yes	Palliative	Home	Yes (FC7)	Male	Husband
P08	Male	60-69	Not recorded	No	Palliative	Home	Yes (FC8)	Female	Wife
P09	Male	60-69	2	Yes	Palliative	Home	Yes (FC9)	Female	Wife

* Specific details of primary cancer site have not been given to protect anonymity but included oropharynx, hypopharynx, tongue, mandible, and parotid gland.

“N/A” = not applicable

Four family carers consented to a joint interview, 3 of whom were female and all were the patients’ spouse (Table 1). The remaining family members either could not be identified or declined participation. Eight HCPs were interviewed, with an equal gender split and their length of time working in healthcare ranged from 15-32 years (Table 2).

**Table 2.** Healthcare Professionals’ Demographic Details (n = 8).

Demographic	Number or range
Gender	Male n = 4 Female n = 4
Age range	38-60 years
Length of healthcare experience	15-32 years
Length of time working with head and neck cancer patients	4-27 years
Current area of work	Specialist Palliative Care n = 2 Community Care n = 2 − General Practitioner n = 1 − Community Nursing n = 1 Head and Neck Cancer Multi-Disciplinary Team members n = 4 − Oncology n = 1 − Surgery n = 2 − Clinical Nurse Specialist n = 1

### Themes

The 2 main barriers identified by HCPs were “lack of consensus about timing of Specialist Palliative Care engagement” and “high stake decisions with uncertainty about treatment outcome.” The main challenge identified by patients and family carers was “lack of preparedness when transitioning from curable to incurable disease.” Additionally, there were 2 overlapping themes from both groups, “uncertainty about meeting psychological needs” and “misconceptions of palliative care.”

### Healthcare Professionals’ Themes

#### Lack of consensus about timing of specialist palliative care engagement

There was uncertainty about the optimum time to commence SPC engagement with recognition that issues could arise when referral was late.

…I have seen people who have clearly been symptomatic for long periods of time, whereby symptom control isn’t possibly as good as it could have been if there was help alongside the way by a specialist. (HCP2)

Engagement with SPC, however, could be perceived as too early in the disease trajectory which was especially challenging for patients still being considered for potentially curative treatment.

…for our locally advanced patients, to offer surgery, but the survival is going to be what, 40% overall survival, and personally I have always been a bit conflicted about whether you should confront these people with palliative type discussions at this stage. (HCP3)

The issue of appropriateness of SPC referral was raised where the treatment intent was palliative but the patient didn’t have overt complex needs.

…I referred them in a manner which said, look, I would rather you were involved earlier. There may be very little for you to do but I think this person should be on your radar…And the letter I got back just didn’t, just didn’t see…Oh well, there’s not much for us to do at this place in time (HCP5)

This raised questions about who was best placed to provide supportive care needs and whether this fell within the SPC remit or was the responsibility of the HNC MDT.

The other group of patients that I often see as well, probably more so than my colleagues in other areas, is that I get referred patients that have undergone curative, or potentially curative treatment for the head and neck cancer, and there doesn’t seem to be anywhere for them to go. (HCP7)

…there’s a need for some support…It might not necessarily be, erm, palliative support, but certainly some, erm, very experienced cancer support…is very good for patients because they’ve got lots of, lots of questions. (HCP8)

Overall, further clarity and consensus to streamline SPC referral processes was welcomed.

…more training would definitely help and trigger what in terms of who to refer to and who may be suitable for each patient would definitely help too. (HCP6)

#### “High stakes” decisions with uncertainty about treatment outcomes

Participants’ reflections conveyed the complexity of decision-making, the unpredictable outcomes and the challenges of advance care planning.

…we take them on for what we hope is good palliative surgery, and they get complications and they end up stuck in hospital for weeks, or they die in hospital, and that in hindsight was a mistake. So, these are high stakes decisions…(HCP3)

This could result in difficulty communicating the relevant information to patients and family carers.

I’m not that convinced that they are really, that the, the sequelae and the side effects of treatment are adequately explained to the patient, that they can really understand what, what it’s going to feel like. (HCP5)

Healthcare professionals could be left wondering how effective their consultations were in terms of information provision.

Sometimes I wonder how well these conversations go, and I look around the room as if to say you know, “How successful was that conversation?” I don’t know. (HCP1)

### Patient and Family Carers Themes

#### Lack of preparedness when transitioning from curative to incurable disease

Both patients and family carers shared experiences where they perceived communication and preparedness was lacking at this critical moment.

In that consultation when I found out I was terminal, I felt that there should have been an additional appointment, or something so that my family could be there and we could discuss it all as a team. (P1*)

…you know, if Dr (doctor’s name) said “go away and absorb the information, I’ll set up for you to come back in a weeks’ time…I’ll arrange for you to see a palliative consultant,” that would have been to my mind…a really good way of managing a very difficult situation. (P9)

Another participant spoke about how unprepared they felt for receiving bad news.

…until yesterday or the day before, I had no idea they were going to say you know, well this is it and you only have a few weeks, I had no idea that was going to happen at all. (P6)

In some situations, there was a desire to have more specific information. One family carer reflected on their wish for details about prognosis.

…He did ask “how long do I have left” and he did say “well I can’t give you years, I could give you months, I could give you 12, I could give you 3. I don’t know.” So, you’re always coming out of those places thinking, oh I wish, but I don’t think they even know themselves to be frank. Then you think, well you see this everyday so come on, you must know something. (FC2)

### Overlapping Themes

#### Uncertainty about meeting psychological needs

Both patients and HCPs recognized that emotional support was an area of great importance.

Psychological support is, is something that’s really necessary, especially if they’ve had quite sort of, like, “mutilating” surgery (HCP5)

It was also recognized as an area of care which could be improved, for both patients and family carers.

Psychologically I find they need a lot of support…sometimes they could do with more. (HCP8)

…I don’t feel that she (my partner) has had enough support, and I do worry about that…I think that’s where you would need the HCPs to be even more supportive to these people, and so they get the additional support they need…(P1*)

It was not clear, however, who should be providing this support. Rather the importance was placed on someone being there to listen.

…just, having somebody to explain or ask you what your fears are (P7)

I wish that when I was diagnosed I could have been put in the direction of a head and neck support team, with people that have been through this. (P2)

#### Misconceptions about palliative care

Societal misconceptions about palliative care representing death and dying were widely reported.

…we’ve told friends that (patient’s name) seen a palliative care consultant and you can see them going…as if palliative care is in the last 6 weeks of your life. (FC9)

Misconceptions were not only isolated to the general public, however, but were also recognized within HCPs.

…the District Nurse who we saw first, I think was quite an experienced district nurse, clearly though the way that most people think about palliative care, is that they’re the people that come in in the last 6 weeks of life…(P9)

Fears about palliative care could mean a reluctance for patients to engage with services.

…sometimes patients are like “no, no, no, I don’t want that,” so we let the GP know we have offered that service, they just don’t want it at the moment. (HCP4)

## Discusssion

Our study findings indicate the main barriers to integrating SPC within routine oncological care relate to the unique complexities of HNC, the decision-making and the uncertainties about treatment outcome. This means it is more challenging to identify the “right” HNC patient at the “right” time who would most benefit from SPC services potentially compared with other cancers. Patients and family carers perceive that increased preparedness for disease transitions and more psychological support are needed. The individual responsibility for the provision of this support wasn’t clearly defined. This study also confirms societal misconceptions about palliative care are also prevalent within a HNC context.

The importance of timely identification of patients who may benefit from SPC is widely recognized. Referrals that are too late can deny patients the full benefit of SPC e.g. timely symptom management and advance care planning. Equally, referrals that are too early may result in patients with few concerns being seen by SPC.^34^ The transition from curative to non-curative disease can be ill-defined and the disease trajectory for HNC patients is especially complex.^35^ A national study identified a cohort of HNC patients, who after initially receiving curative treatment, were quickly recognized to have residual or recurrent cancer and required a palliative care focus.^36^ Hence, there may be specific periods when the patient might benefit from a targeted SPC input focused on symptom control, even although the intent of treatment is curative.^37^ It is not feasible, however, for every HNC patient being treated with palliative intent to receive input from a relatively scare specialist resource.

This links with Quill and Abernethy’s “coordinated palliative care model” where contributions from both specialists and non-specialists in palliative care are valued.^38^ The model distinguishes primary palliative care skills (skills which all clinicians should have) from specialist skills (those for managing more complex, challenging situations) but enables both to work together in a collaborative manner.^38^ The primary care or treating specialist would lead the initial palliative care, involve the SPC team for complex or intractable issues, and then continue the ongoing care if, and when, the issues were resolved. The European Association for Palliative Care (EAPC) has provided recommended levels of education in palliative care to support this model.^39^

Despite national recommendations that all core HNC MDT members should have advanced communication skills,^28^ issues were identified relating to the information provision and goals of care. This is similar to another study exploring the communication of prognostication information to HNC patients.^40^ Issues identified related to medical jargon, paternalism and the omission of specific prognostic information.^40^ Additionally, HNC patients can have a reluctance to engage with advance care planning due to their focus on treatments which increase the longevity of life^41^ and differences in preferences about the level of information they desire.^42^

A systematic review reported that factors promoting good partnership working between specialists and non-specialists in palliative care included: clear definition of roles and responsibilities; good communication; shared learning and education; appropriate, timely SPC access and coordinated care.^43^ Our study findings would suggest clarity about roles for providing psychological support is important. Additionally, in the context of complex HNC decisions, uncertain treatment outcomes and emerging immunotherapy treatments,^44^ another area of focus and collaboration would be defining and effectively communicating goals of care.

The optimal way to “incorporate palliative care in the multidisciplinary management of patients with high risk squamous cell cancer of the head and neck” remains unclear.^45^ Internationally, to help gain consensus on who should be referred and the optimal timing of SPC out-patient referral, a Delphi study was conducted.^34^ This defined 11 major needs- and timing-based referral criteria. In view of the less predictable disease trajectory, criteria such as these need further validation within the HNC remit to see if they provide timely and appropriate SPC referrals. Additionally, although there are a number of models promoting palliative care integration within oncological care, including time-, provider-, issue-, or system-based models,^12^ it is unclear which are the most appropriate for HNC patients. Investment into research funding is required to evaluate specific interventions which promote high quality care and good communication. As HNC is associated with “aggressive” interventions at the end-of-life,^46,47^ a focus on cost-effective use of healthcare resources would also be pertinent.

### Strengths and Limitations

This study has several strengths. Firstly, data from patients, family carers, and HCPs enabled multiple perspectives to be obtained, bringing breadth and depth to the study. The researcher conducting the interviews had a psychology background potentially enabling a more open approach to both positive and negative care experiences. Although previous studies have explored challenges to integrating palliative care, none have specifically focused within a HNC population. Many HNC qualitative studies have assessed issues earlier in their disease trajectory.^8,48^ By engaging with advanced HNC patients, they represent a “hard to reach” group.

There were limitations to the study. Firstly, we did not recruit any patients who had been treated with curative intent but were recognized to be “high risk” for recurrence. There may have been a degree of healthcare professional “gatekeeping” and a reluctance to consider those who were not already linked into SPC services in case participation potentially caused distress. Their viewpoint could have helped identify additional challenges faced earlier in the illness. Secondly, due to difficulties with verbal communication, 1 patient interview was very short. Limiting the study to only those who were verbally articulate, however, did not seem ethically appropriate. Thirdly, member checking of the transcripts was not deemed to be practical due to the advancing illness and the limited time available for the HCPs. Finally, the study was conducted within 1 healthcare system and further research would be valuable to explore barriers within different countries and systems.

## Conclusion

HNC reflects an illness with a less predictable disease trajectory, where highly complex decisions are made and treatment outcomes can be less certain. A specific focus needs to be given to the optimal way to initiate SPC referrals which may not be in keeping with those used for the wider cancer population. Clearer ways to effectively communicate the goals of care are required potentially adopting a collaborative approach between SPC and the wider HNC MDT earlier in the disease trajectory.

## Supplemental Material

Supplemental Material, Supplementary_file_1_Interview_schedule_9TH_DEC_2019 - A Qualitative Study Exploring Patient, Family Carer and Healthcare Professionals’ Direct Experiences and Barriers to Providing and Integrating Palliative Care for Advanced Head and Neck Cancer Click here for additional data file. Supplemental Material, Supplementary_file_1_Interview_schedule_9TH_DEC_2019 for A Qualitative Study Exploring Patient, Family Carer and Healthcare Professionals’ Direct Experiences and Barriers to Providing and Integrating Palliative Care for Advanced Head and Neck Cancer by Catriona Rachel Mayland, Hannah C. Doughty, Simon N. Rogers, Anna Gola, Stephen Mason, Cathy Hubbert, Dominic Macareavy and Barbara A. Jack in Journal of Palliative Care

## Abbreviations

HCP: healthcare professionals
HNC: head and neck cancer
MDT: Multi-disciplinary Team
SPC: Specialist Palliative Care
UK: United Kingdom
