# Supplementary material for: A Qualitative Study Exploring Patient, Family Carer and Healthcare Professionals’ Direct Experiences and Barriers to Providing and Integrating Palliative Care for Advanced Head and Neck Cancer
Source: J Palliat Care. 2020 Sep 15;36(2):121–9. doi: 10.1177/0825859720957817 (PMC7961626; doi:10.1177/0825859720957817)
Supplement: Supplemental Material, Supplementary_file_1_Interview_schedule_9TH_DEC_2019 - A Qualitative Study Exploring Patient, Family Carer and Healthcare Professionals’ Direct Experiences and Barriers to Providing and Integrating Palliative Care for Advanced Head and Neck Cancer [file Supplementary_file_1_Interview_schedule_9TH_DEC_2019.docx]

**Supplementary file 1: Interview schedules – with questions relevant to this study**

**Outline of interview schedule – Healthcare professional (HCP)**

*We want HCPs to talk about their experience of providing care to patients with advanced head and neck cancer in the recent months/years. We want HCPs to discuss any challenges that they have faced with regard when providing palliative care to patients and any ways they feel that services may need to be changed/improved to improve the care that patients and families receive.*

Exploratory questions within the interview include:

- Can you tell me about your experience caring for patients with advanced head and neck cancer?
- Can you think of a particular situation where care delivery went well?
- Have you experienced any challenges in terms of care provision?
- In terms of palliative care, are there ways you could see services working differently that could be beneficial?
- What models of care do you think might work?
- Are there any specific timeframes for the initiation of Specialist palliative Care (SPC) for patients with H&N cancer?
- What are your thoughts about the timing of appropriate referral to SPC?
- What are your thoughts and feelings about earlier involvement of SPC?
- Would you perceive other ways of integrating palliative care which would better meet the needs of patients?

**Outline of interview schedule – Patients and family carer**

*We want patients and family carers to talk about their experience of care in the recent months, and discuss any challenges that they have faced with regard to their care (including palliative care, where appropriate) and any ways they feel may improve the care that themselves and other patients receive.*

Exploratory questions within the interview may include:

- What has been your experience of having head and neck cancer?
- Have there been any specific challenges and difficulties?
- What are your perceptions about the care and service delivery you have received? What has worked well? Have there been any specific issues?
- Are there ways you could see services working differently that would be beneficial?
- What are your experiences of palliative care (where appropriate, and how this linked to their overall care)?
